# Anatomically Guided Non-Viral CRISPR/Cas9 Delivery in the Eye: Overcoming Barriers for Precision Gene Therapy

**DOI:** 10.3390/pharmaceutics18030282

**Published:** 2026-02-24

**Authors:** Zhixiang Hua, Yang Shen, Xingtao Zhou

**Affiliations:** 1 Eye Institute and Department of Ophthalmology, Eye & ENT Hospital, Fudan University, Shanghai 200031, China; 19211260005@fudan.edu.cn; 2 NHC Key Laboratory of Myopia (Fudan University), Key Laboratory of Myopia, Chinese Academy of Medical Sciences, Shanghai 200031, China; 3 Shanghai Engineering Research Centre of Laser and Autostereoscopic 3D for Vision Care, Shanghai 200031, China; 4 Shanghai Research Centre of Ophthalmology and Optometry, Shanghai 200031, China

**Keywords:** CRISPR/Cas9, non-viral delivery, physiological barriers, anatomical barriers, ocular gene therapy

## Abstract

Background/Objectives: While CRISPR/Cas9 technology offers a revolutionary approach for correcting genetic ocular blindness, efficient and safe delivery remains the primary bottleneck. Traditional viral vectors, despite their efficacy, face challenges regarding cargo size limitations and potential genomic integration risks. Non-viral vectors offer distinct comparative advantages, including large cargo capacity for diverse CRISPR tools and transient expression to minimize off-target effects, but must overcome the eye’s formidable static and dynamic barriers, specifically the corneal epithelium, vitreous humor, and the inner limiting membrane. In this review, we present an anatomically guided framework for non-viral CRISPR/Cas9 delivery, mapping engineering strategies to specific ocular tissue targets. We first delineate the mechanisms of key physiological barriers, including the corneal stroma, aqueous humor circulation, and the vitreous–retina interface. Subsequently, we critically evaluate the latest advancements in non-viral platforms, such as pH-responsive lipid nanoparticles and engineered virus-like particles. The core focus of this review is on site-specific breakthrough strategies: from utilizing mucoadhesive polymers to counteract tear clearance in the cornea to exploiting specialized administration routes, such as suprachoroidal space and subretinal injection, to bypass retinal barriers, and deep-penetrating intravitreal carriers for targeting the photoreceptor-RPE complex. By integrating material science with precise administration routes, this review highlights feasible translational pathways for next-generation, carrier-free, or biomimetic ocular gene editing therapies.

## 1. Introduction

### 1.1. Functionality and Overview of the CRISPR/Cas9 System

Since its development and application in gene editing, the CRISPR/Cas9 system has revolutionized genetic engineering due to its high efficiency, specificity, and ease of use. It has rapidly become one of the most representative and widely used tools in the field of genome editing [1,2]. This system mainly involves a guide RNA (gRNA) that guides the Cas9 nuclease to the target DNA sequence and induces a site-specific double-strand break (DSB) [3]. After DSB generation, cellular DNA repair mainly occurs through two pathways, including non-homologous end joining (NHEJ) and homology-directed repair (HDR) [3,4]. NHEJ is an efficient but error-prone repair process and usually introduces small insertions or deletions (indels), resulting in gene disruption or knockout. HDR, by contrast, can achieve precise gene correction, insertion, or replacement when an exogenous homologous repair template is provided [3]. Based on these mechanisms, CRISPR/Cas9 technology offers a powerful platform for correcting pathogenic mutations at the genetic level, thereby enabling therapeutic interventions for a variety of inherited diseases and showing great promise for clinical translation [5].

### 1.2. The Significance of CRISPR/Cas9 in Treating Ocular Diseases

Inherited ocular diseases are a major cause of visual impairment and blindness worldwide and show high genetic heterogeneity. Disorders such as retinitis pigmentosa (RP), Leber congenital amaurosis (LCA), and different types of corneal dystrophy are caused by single- or multiple-gene mutations [6,7]. These mutations affect the function or survival of retinal or corneal cells. As a result, visual function is progressively impaired, leading to a marked reduction in patients’ quality of life [6,7].

Currently, there are multiple clinical studies underway exploring the application of CRISPR/Cas9 technology for the treatment of ocular diseases (Table 1), offering a novel and precise approach for genetic intervention. The eye, due to its relatively enclosed anatomical structure and partial immune privilege, is particularly well-suited for gene therapy and genome editing [8]. However, traditional gene augmentation faces intrinsic limitations, particularly in dominant-negative disorders where the toxic mutant protein remains active despite gene supplementation. CRISPR/Cas9 overcomes this bottleneck by enabling the direct silencing or correction of the endogenous pathogenic allele, offering a definitive therapeutic solution unavailable to augmentation strategies [9,10].

### 1.3. The Role of Non-Viral Delivery Systems for CRISPR/Cas9 in Ocular Gene Editing

Viral vectors, particularly adeno-associated viruses (AAVs), have been widely used to deliver CRISPR/Cas9 components and have shown clinical benefits in ocular diseases. However, their application is inherently constrained by fundamental bottlenecks, including packaging limits that restrict the use of advanced CRISPR tools and safety concerns related to persistent viral expression [5,6,7]. As elaborated in Section 2, these limitations create a significant gap in current therapeutic capabilities.

To bridge this gap, increasing efforts have been directed toward non-viral delivery strategies for CRISPR/Cas9. The primary rationale for adopting non-viral systems lies in their ability to offer expanded cargo capacity and transient expression profiles, alongside generally lower immunogenicity. These features make them suitable candidates for in vivo genome editing applications [5,11,12]. In practice, however, efficient non-viral delivery in ocular tissues remains difficult. Physical and biological barriers, such as the corneal epithelium and the blood–retina barrier, strongly restrict the transport of gene-editing components.

For this reason, there is a clear need for non-viral delivery platforms that can balance efficiency and safety while maintaining sufficient targeting accuracy. It should deliver Cas9 protein, single-guide RNA, or nucleic acid forms encoding Cas9 to specific cell types within the eye [13]. In this review, we present an anatomically guided framework for non-viral CRISPR/Cas9 delivery. By integrating emerging non-viral nanocarriers with ocular physiological barriers, we provide insights into selecting suitable vectors and administration routes, thereby optimizing gene editing therapies for both the anterior and posterior segments.

## 2. Limitations of Viral Delivery and Advantages of Non-Viral Systems

Gene editing using CRISPR/Cas9 is being explored for inherited ocular diseases, but the outcome largely depends on how CRISPR components are delivered to ocular tissues. A well-known example is AAV-based therapy for RPE65-associated Leber congenital amaurosis (LCA), which has reached the clinic and illustrates what efficient ocular delivery can achieve [14,15]. For CRISPR, however, delivery needs are different. In many cases, the system should be efficient but also tightly controlled, especially to limit unwanted exposure and long-term risks.

### 2.1. Limitations of Viral Delivery Strategies

AAV is one of the most frequently used viral vectors in the eye, yet several practical issues limit its use for CRISPR/Cas9 delivery. One major concern is immunogenicity. AAV is generally considered less immunogenic than some other viral vectors, but immune responses can still occur. Capsid-related immunity, and in particular sustained expression of a bacterial Cas9 protein, may trigger adaptive immune responses [10,16]. In ocular settings, this can reduce editing efficiency and therapeutic benefit, and in some cases may lead to inflammation that threatens visual function [8]. The problem becomes more relevant in patients who need repeat dosing or who already carry neutralizing antibodies. Another limitation is payload size. AAV has a relatively small packaging capacity (around 4.7 kb) [17,18]. The SpCas9 coding region itself is close to this limit (~4.2 kb), leaving little space for promoters, gRNA expression cassettes, and other regulatory elements. As a result, vector design often becomes complicated (for example, dual-vector strategies), which can reduce overall feasibility and consistency [10,19]. Long-term expression is also a concern. AAV commonly remains episomal and is considered to have a low integration rate, but prolonged Cas9 expression is not always desirable for genome editing. Extended exposure increases the chance of accumulating off-target events over time, and it complicates safety assessment. For these reasons, the long-term genomic safety of viral-mediated CRISPR delivery still needs careful evaluation in clinical contexts [16,20].

### 2.2. Advantages of Non-Viral Delivery Systems

Non-viral delivery systems provide alternative options for ocular CRISPR/Cas9 applications and may address several limitations seen with viral vectors. First, many non-viral carriers are built from biodegradable and biocompatible materials, which usually show low immunogenicity. Given the relatively immune-privileged nature of the eye, reducing immune activation is particularly important to avoid inflammatory damage [16,21]. Second, non-viral systems place fewer limitations on cargo size, which allows delivery of larger CRISPR formats, such as Cas9 mRNA or Cas9 ribonucleoprotein (RNP) complexes, without extensive vector optimization. This flexibility makes it easier to adjust editing strategies and combine different components when needed [21]. Third, non-viral delivery typically avoids insertional integration risks, which helps reduce concerns related to insertional mutagenesis and potential oncogenic events [22].

In addition, non-viral materials can be engineered to control release behavior. This allows transient delivery or short-term expression, which is often preferred for CRISPR because it can lower off-target risk and reduce long-term toxicity [13,16,21]. Despite these advantages, current non-viral systems still face challenges regarding in vivo transfection efficiency and stability, falling short compared to viral vectors [23]. In the ocular context, these limitations are associated with the eye’s unique anatomical barriers, which are discussed in detail in the following section.

## 3. Ocular Physiological Barriers to Non-Viral Delivery

Non-viral delivery systems are considered useful for ocular CRISPR/Cas9 applications, but their performance in the eye is still limited. This is mainly because the eye has several anatomical and physiological features that restrict the movement and retention of exogenous materials. These barriers protect ocular tissues under normal conditions, but they also reduce delivery efficiency.

### 3.1. Barrier Effects of the Ocular Surface

The static barrier at the ocular surface mainly refers to the cornea. Its multilayered anatomical structure restricts the penetration of delivery systems across tissues. The cornea consists of several distinct layers, including the epithelium, Bowman’s layer, stroma, Descemet’s membrane, and endothelium [24,25]. The epithelial layer contains abundant lipids and tight junctions, limiting the passage of hydrophilic molecules. In contrast, the stroma is mainly composed of collagen and is highly hydrophilic, thereby restricting the diffusion of lipophilic substances [26,27]. Because of these opposing properties, effective delivery across the cornea usually requires carriers with both hydrophilic and lipophilic characteristics [27].

In addition to these structural barriers, dynamic processes at the ocular surface further limit delivery efficiency. After topical administration, formulations are rapidly diluted by tear fluid and removed from the ocular surface through blinking. Because tear fluid is rapidly renewed, residence time on the corneal surface is short, and penetration into corneal tissues is often limited [28]. In addition, drainage through the nasolacrimal system lowers local ocular availability and can expose the drug to the nasal cavity and systemic circulation [27,29]. Together, rapid dynamic clearance and static penetration barriers pose a major challenge for conventional topical ocular delivery.

### 3.2. Barrier Effects Within the Eye

#### 3.2.1. Vitreous and Inner Limiting Membrane (ILM)

The vitreous is a gel-like matrix located between the lens and the retina and contains mainly water, collagen fibers, and hyaluronic acid [30]. Because of its viscous nature, diffusion of nanoparticles in the vitreous cavity is relatively slow. As particle size increases, movement becomes more restricted, and carriers tend to remain near the injection site rather than reaching retinal cells or the retinal pigment epithelium (RPE) [27,31]. The inner limiting membrane (ILM) lies between the vitreous and the retinal tissue. It is a basement membrane composed of extracellular matrix (ECM) proteins produced by retinal cells [32]. Although the exact size threshold for penetration varies across species and has not been clearly defined, the ILM shows a strong size exclusion effect. While small molecules can diffuse passively, large nanoscale carriers are largely restricted [33]. As a result, materials delivered by intravitreal (IVT) injection often accumulate on the ILM surface and show limited penetration into deeper retinal layers [34]. Improving transport beyond the vitreous and ILM therefore depends largely on adjusting particle size and surface characteristics to allow better diffusion and passage across these barriers.

#### 3.2.2. Blood–Retinal Barrier (BRB)

The blood–retinal barrier (BRB) restricts the movement of molecules within the eye. It is formed by tight junctions between retinal vascular endothelial cells and retinal pigment epithelial (RPE) cells, which reduce the passage of blood-borne substances into retinal neural tissue [35,36,37]. At the same time, the BRB restricts the entry of many therapeutic agents, including CRISPR/Cas9 components, into retinal target cells after systemic or some intraocular delivery routes. To cross the BRB, delivery systems usually require specific design features. In some cases, nanoparticles with appropriate surface ligands or small particle sizes can cross the BRB via receptor-mediated uptake or transcellular transport [28]. For instance, nanoparticles functionalized with transferrin have been shown to cross retinal barriers via transferrin receptor-mediated endocytosis, effectively delivering therapeutic plasmids to retinal tissues [38]. Currently, intravitreal and subretinal injections are the main routes used to deliver therapeutics to the posterior segment of the eye [39,40].

#### 3.2.3. Aqueous Humor Circulation

Aqueous humor is produced by the ciliary body, moves through the posterior and anterior chambers, and is drained via the trabecular meshwork [41]. This circulation helps maintain intraocular pressure and supplies nutrients to avascular tissues. At the same time, it removes exogenous materials from the anterior chamber at a relatively fast rate [42]. As a result, CRISPR components delivered to the anterior segment often show short residence times in tissues such as the corneal endothelium, trabecular meshwork, or iris, which limits effective exposure and delivery efficiency.

## 4. Non-Viral CRISPR/Cas9 Delivery Systems

**Figure 1:**
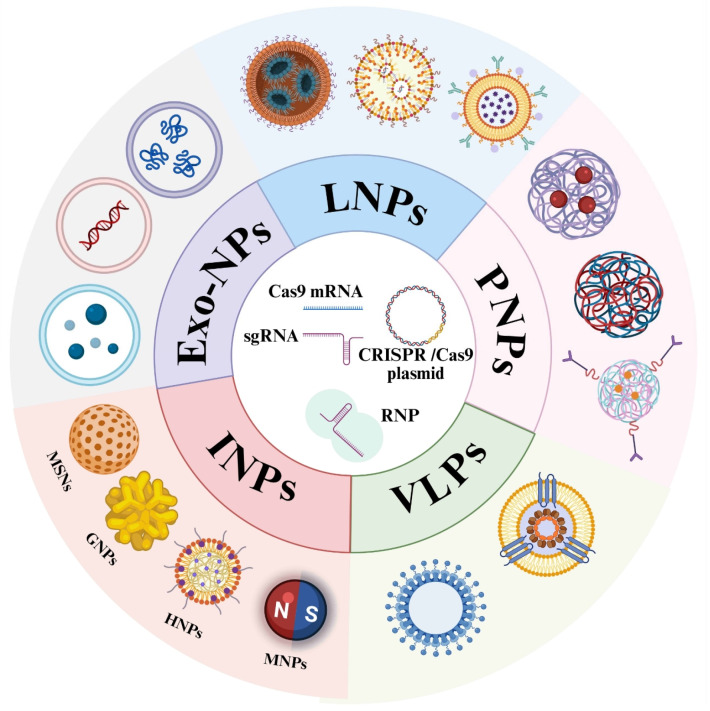
Schematic classification of non-viral delivery systems for ocular CRISPR/Cas9 therapy. Created in BioRender. Hua, Z. (2026) https://BioRender.com/jsg54vi.

To address the physiological barriers described above, various non-viral delivery strategies have been utilized. In this section, we provide a concise overview of five major classes of non-viral vectors, lipid nanoparticles (LNPs), polymer nanoparticles (PNPs), inorganic nanoparticles, virus-like particles (VLPs), and exosome-based systems, as illustrated in Figure 1. A systematic comparison of these platforms, summarizing their key advantages and limitations, is provided in Table 2.

### 4.1. Lipid Nanoparticles (LNPs)

Lipid nanoparticles (LNPs) are widely used as a non-viral vector and have progressed toward clinical application. They are formed by the self-assembly of several lipid components, including ionizable lipids, helper lipids such as cholesterol, PEGylated lipids, and neutral lipids [43,44]. The core advantage of LNPs lies in the pH-responsive behavior of ionizable lipids: they remain largely neutral at physiological pH, which helps reduce systemic toxicity, but become protonated in the acidic environment of endosomes. This change is associated with disruption of the endosomal membrane and facilitates the release of CRISPR components into the cytoplasm [43].

When LNPs are used to deliver short-lived Cas9 mRNA or ribonucleoprotein (RNP) complexes, long-term persistence of exogenous genes and genomic integration can be avoided. This reduces the accumulation of off-target effects and lowers the risk of immune rejection, providing a favorable safety window for ocular gene therapy. In the treatment of eye diseases, the nanoscale size of LNPs and their tissue penetration capability have enabled delivery to multiple anatomical targets [44]. For corneal disorders, newly developed LNP platforms have been shown to penetrate the corneal stroma and deliver Cas9 RNP together with donor DNA to target cells [45]. For retinal neovascular diseases, dynamic covalent LNP systems (LNP-ABC) have been used for the co-delivery of Cas9 mRNA and VEGFA-targeting sgRNA, highlighting the applicability of LNPs as carriers for precision ocular gene therapy [46].

However, significant challenges remain for widespread clinical adoption. Stability is a primary concern; commercial formulations often require storage below −20 °C, as transfection efficiency can drop by over 50% even after short-term storage at 4 °C [47]. In addition, LNPs may also induce serum complement activation, leading to a non-IgE mediated hypersensitivity reaction known as complement activation-related pseudoallergy (CARPA), which can ultimately result in anaphylaxis [48,49]. Finally, manufacturing scalability, hindered by batch-to-batch heterogeneity, can significantly impact therapeutic consistency [50].

### 4.2. Polymer Nanoparticles (PNPs)

Polymer nanoparticles (PNPs) are another important class of non-viral delivery systems. Because of their good biocompatibility, biodegradability, and ease of chemical modification, they have attracted broad interest for ocular CRISPR/Cas9 delivery [51]. Common polymers used for CRISPR/Cas9 delivery include polyethyleneimine (PEI), poly(lactic-co-glycolic acid) (PLGA), polycaprolactone (PCL), dendrimers, fluorinated polymers and biological polymers such as cell-penetrating peptides (CPPs) [51,52].

Compared with lipid nanoparticles, a main advantage of PNPs is their potential for functional modification. By conjugating specific ligands, PNPs can enhance the enrichment of CRISPR components in the posterior segment of the eye. This helps reduce off-target effects in non-target cells and improves therapeutic efficiency while lowering systemic toxicity [53,54]. In addition, novel fluorinated polymers have shown progress in ocular mRNA delivery. mRNA nanoparticles formed from these materials can bind serum albumin and promote cellular uptake through scavenger receptor-mediated endocytosis [55]. In choroidal neovascularization models, fluoropolymer-mediated delivery of Cas9 and base editor mRNA effectively downregulated VEGFA expression. These results support their use as efficient carriers for ocular gene-editing applications [55].

However, clinical translation is constrained by safety and manufacturing factors. Cationic PNPs typically exhibit cytotoxicity linked to membrane disruption [56]. Additionally, the structural modifications required for versatility complicate large scale production and batch-to-batch reproducibility [57].

### 4.3. Inorganic Nanoparticles (INPs)

Inorganic nanoparticles have been used as non-viral carriers for CRISPR/Cas9 delivery in ocular research. These materials differ in size and surface properties, which allows some flexibility in delivery design. Several types of inorganic nanoparticles have been tested, including gold nanoparticles, magnetic nanoparticles, mesoporous silica nanoparticles, and hybrid systems [23,58].

Gold nanoparticles (GNPs) have been studied as carriers for gene delivery because of their general biocompatibility and low cytotoxicity [59]. Their surfaces can be modified through electrostatic or covalent interactions with Cas9 protein, sgRNA, or Cas9-encoding DNA or mRNA, which supports cellular uptake of CRISPR components [60,61]. In some studies, external light stimulation has been applied to assist membrane permeabilization and intracellular entry. GNPs have also been used for imaging-based tracking during delivery processes [62,63,64].

Magnetic nanoparticles (MNPs), such as superparamagnetic iron oxide particles, have been explored as delivery carriers because they respond to external magnetic fields. After loading with CRISPR components, their movement can be influenced by magnetic guidance in a non-contact manner [65,66,67]. This property has been discussed as a way to assist delivery under ocular fluid conditions. In addition, superparamagnetic iron oxide nanoparticles can act as contrast agents for magnetic resonance imaging (MRI), allowing visualization of particle distribution in vivo [68,69].

Mesoporous silica nanoparticles (MSNs) have been studied as carriers for gene delivery because of their porous structure and large surface area [70,71]. These pores allow loading of large biomolecules, such as Cas9 ribonucleoprotein complexes or plasmid DNA, and can help protect them from degradation. In addition, the surface of MSNs can be modified with different functional molecules, including cell-penetrating peptides or targeting ligands, which supports further investigation of their use as delivery carriers [70,72].

Hybrid nanoparticles (HNPs) are constructed by combining inorganic materials with organic polymers. In these systems, CRISPR plasmids are physically encapsulated within lipid–polymer networks rather than complexed through cationic components. This design helps reduce cytotoxicity and particle aggregation that are often associated with electrostatic complexes. In addition, surface conjugation with intercellular adhesion molecule-1 (ICAM-1) antibodies has been used to improve localization to specific tissues [73]. Inorganic nanoparticles offer distinct advantages for CRISPR delivery in gene therapy. However, many types are non-biodegradable, posing risks of long-term ocular accumulation [74]. Additionally, inconsistent reporting of physicochemical properties makes it difficult to reproduce results or benchmark outcomes [75]. With most studies limited to cellular or small animal models, clinical translation remains a challenge [75,76]. Overcoming these hurdles requires harmonized characterization standards, extensive large-animal studies, and detailed monitoring of biodistribution and gene-editing outcomes in early human trials [77].

### 4.4. Virus-like Particles (VLPs)

Virus-like particles (VLPs) are non-viral delivery systems that mimic the structure of viruses. They are assembled from viral structural proteins but do not contain viral genomes. As a result, the risk of replication is avoided, while efficient cellular entry and nuclear delivery are retained [78,79]. To address the challenge of ribonucleoprotein (RNP) loading, engineered aptamer–tag strategies have been developed. This approach enables specific co-assembly of Cas9 RNPs with VLPs. Only fully active editing components are packaged, which improves both loading specificity and delivery accuracy [80,81].

Functionally, VLP-based delivery shows both high efficiency and low off-target activity. When preassembled RNPs are delivered by VLPs, Cas9 expression is transient. This short intracellular residence time reduces the risk of off-target effects [82]. In addition, VLPs enter the nucleus efficiently through biomimetic mechanisms. Their gene-editing efficiency has been reported to be comparable to that of AAV and lentiviral vectors and higher than that of lipid nanoparticles. At the same time, off-target mutation frequencies are lower than those observed with electroporation-based delivery [78,83]. Moreover, VLPs have shown potential in delivering enhanced-deletion Cas9 (EDCas9) constructs for targeting splicing defects in genetic eye diseases [84,85].

However, distinct challenges exist for their clinical application. First, immunogenicity remains a potential issue, as viral capsid proteins may still trigger host immune responses [86]. Second, cargo capacity is restricted by the rigid capsid structure, limiting the packaging of larger CRISPR systems [87]. Third, production and purification are complex, often requiring stringent optimization to ensure particle stability and uniformity [87,88].

### 4.5. Exosome-Based Nanoparticles (Exo-NPs)

Exosomes are endogenous nanoscale vesicles secreted by cells, with sizes ranging from approximately 30 to 150 nm. They can carry various bioactive molecules and play an important role in intercellular communication [89]. Compared with synthetic carriers, their most notable feature is their natural homing ability. Exosomes display specific integrins and membrane proteins on their surface, which confer spontaneous affinity toward certain tissues [90]. Because of their low immunogenicity, efficient membrane-crossing capability, and high stability, exosomes are considered suitable natural carriers for gene delivery. These properties are particularly relevant for ocular disease treatment, where exosomes have shown distinct advantages [91,92,93,94].

Several proof-of-concept studies have validated CRISPR/Cas9 delivery via exosome-based platforms in the eye. For example, a hybrid nanoparticle system combining exosomes and liposomes, modified with the nucleic acid aptamer AS1411, was designed for targeted delivery of CRISPR/Cas9 to suppress choroidal neovascularization (CNV) [95]. By merging the structural stability of liposomes with the biocompatibility and targeting properties of exosomes, this strategy provides a foundation for developing safe, potentially re-dosable ocular gene editing systems.

However, several challenges hinder the clinical translation of EVs. First, safety risks associated with the EV source are a major concern; for instance, tumor-derived EVs may facilitate metastasis, and co-isolated endogenous macromolecules could trigger unintended biological effects [96]. Second, in vivo efficacy is limited by rapid immune clearance and unstable targeting capabilities, which may lead to off-target effects [97]. Finally, low payload capacity remains a technical bottleneck, prompting the development of hybrid systems to enhance the loading efficiency of CRISPR RNPs [98].

## 5. Anatomy-Based Targeted Therapeutic Strategies

**Figure 2:**
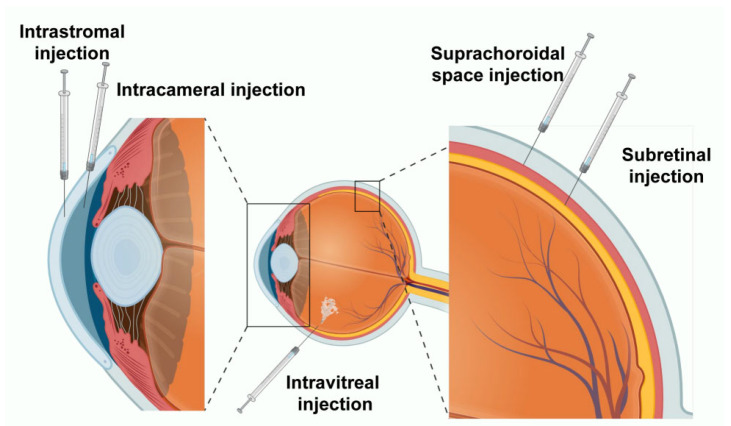
Schematic representation of ocular anatomical barriers and the five primary non-viral gene delivery routes targeting the anterior and posterior segments. Created in BioRender. Hua, Z. (2026) https://BioRender.com/7ktpwmw.

Ocular tissues exhibit significant heterogeneity in anatomical structure, cellular composition, and physiological barriers, which determines that a single delivery modality cannot fulfill the diverse requirements for different lesion sites in terms of delivery efficiency and safety boundaries. Therefore, this section outlines the key delivery barriers across different ocular tissues based on anatomical location, and summarizes corresponding engineering optimization strategies and feasible translational approaches by integrating the material characteristics of non-viral delivery systems and administration routes (Figure 2).

### 5.1. Full-Thickness Corneal Strategies: Epithelium, Stroma, and Endothelium

Gene therapy targeting the cornea includes both infectious diseases (e.g., herpes simplex keratitis, HSK) and various hereditary corneal dystrophies. As target cells are distributed across different corneal layers, the design of non-viral delivery systems must match the depth of the target tissue: superficial layers require strategies to extend ocular surface retention and enhance permeability, while deeper layers often rely on physical bypass approaches such as local injection, with priority given to tissue safety and biocompatibility.

#### 5.1.1. Corneal Epithelium: Mucoadhesive Strategies to Counteract Dynamic Clearance

Therapeutic needs in the corneal epithelium are mainly found in diseases such as Meesmann epithelial corneal dystrophy (MECD), where CRISPR-mediated allele-specific intervention may be used to selectively silence mutant keratin genes (e.g., KRT12) to restore epithelial structure and function [99,100,101]. Delivery barriers at this level are not due to tissue depth but stem from two main factors: (1) the dynamic clearance of the ocular surface significantly shortens carrier contact time; (2) tight junctions in epithelial cells form a physical barrier that limits trans-layer permeation of delivery systems. In this context, polymer nanoparticles (PNPs) with mucoadhesive properties are considered one of the most suitable non-invasive delivery platforms [51]. For example, chitosan, with its positive charge, can interact electrostatically with negatively charged corneal mucins to enhance surface adhesion and resist tear washout [102]. More importantly, chitosan has also been reported to transiently modulate tight junction permeability, facilitating paracellular transport of Cas9 mRNA or RNP-loaded carriers across the epithelium [103,104]. Overall, this synergistic strategy of “adhesion prolongation and permeability enhancement” holds promise for non-invasive gene repair of the epithelium without compromising corneal integrity.

#### 5.1.2. Corneal Stroma: Dual Targeting of Infection and Genetic Disorders with Physical Bypass

The corneal stroma accounts for about 90% of corneal thickness and is the main structure responsible for corneal transparency and refractive power [105]. Gene therapy needs in this region mainly fall into two categories: inherited stromal dystrophies and infectious diseases. For inherited disorders, corneal stromal dystrophies often involve misfolding and deposition of stromal proteins. Typical examples include mutations in the CHST6 or TGFBI genes [106,107]. For infectious diseases, herpes simplex virus keratitis (HSK) is one of the leading causes of corneal blindness. Clinically, HSK presents as recurrent corneal inflammation. However, the underlying cause of recurrence is the persistence of HSV-1 in nerve fibers or stromal cells within the corneal stroma [108]. Although both disease types require delivery across deep stromal barriers, the gene-editing strategy for HSK differs fundamentally from that used for inherited disorders. The goal is not to repair the host genome. Instead, it is to specifically target and efficiently cleave the viral genome to prevent recurrence [109].

Intrastromal injection provides a key physical bypass of corneal physiological barriers. Through microsurgical procedures, LNPs or VLPs can be directly injected into the stromal layer. This approach ensures that a high concentration of gene-editing tools is delivered directly to the lesion site [110,111]. Studies have shown that this strategy can correct metabolic defects and eliminate the HSK viral reservoir without damaging host nerve function [109,110,112]. Although this technique requires precise operation and can cause transient corneal edema, this form of direct delivery offers an irreplaceable clinical option for treating refractory corneal diseases.

#### 5.1.3. Corneal Endothelium: Anterior Chamber Route and Zero-Toxicity Delivery

Corneal endothelial cells (CECs) are located in the innermost layer of the cornea. They maintain corneal dehydration and transparency through a pump-leak mechanism [113]. After adulthood, CECs show very limited regenerative capacity. If cell density falls below a critical threshold due to treatment-related injury, irreversible visual impairment and bullous keratopathy can occur [113,114,115]. For this reason, delivery strategies targeting the corneal endothelium must overcome deep anatomical barriers while prioritizing cellular safety. At present, intracameral injection (IC) is considered the most suitable route for corneal endothelial gene therapy. This approach exposes delivery vectors directly to the endothelial surface and achieves high local bioavailability. When combined with magnetic guidance or surface charge–optimized LNP or AAV vectors, efficient transfection can be achieved with minimal cytotoxicity [116,117]. Fuchs endothelial corneal dystrophy (FECD) is a common inherited endothelial disorder. Its pathology is mainly associated with mutations in genes such as COL8A2, TCF4, or SLC4A11, which lead to severe endoplasmic reticulum stress and endothelial cell apoptosis [118,119,120,121]. In a mouse model of COL8A2 mutation–induced FECD, intracameral delivery of the CRISPR/Cas9 system successfully reduced mutant gene expression and restored endothelial pump function. These results support the potential of anterior chamber delivery for treating corneal endothelial diseases [122].

Overall, effective treatment of corneal diseases requires the integration of non-viral carrier properties, such as the adhesive characteristics of polymer nanoparticles, with appropriate delivery routes, including intrastromal and intracameral injection. This combined strategy enables efficient and precise delivery of CRISPR/Cas systems to the anterior segment of the eye.

### 5.2. Trabecular Meshwork (TM) Strategy

The trabecular meshwork (TM) is located at the iridocorneal angle of the anterior chamber. It is the main filtration pathway for aqueous humor outflow. Its function directly determines intraocular pressure (IOP) homeostasis. In primary open-angle glaucoma (POAG), elevated IOP is often linked to mutations in the MYOC (myocilin) gene. These mutations lead to abnormal protein accumulation and TM cell death [123]. Therefore, targeted knockout of mutant MYOC using the CRISPR/Cas system is considered a potential strategy for permanent treatment of this type of glaucoma [123,124]. However, delivery to the TM is strongly affected by aqueous humor dynamics. As the TM lies along the outflow pathway, continuous fluid flow can rapidly remove delivery vectors before they are taken up by target cells. To address this issue, a combined strategy of intracameral injection (IC) and carrier engineering has been adopted. Intracameral injection allows CRISPR components to bypass the corneal barrier and directly access the region surrounding TM cells [123].

On this basis, additional engineering approaches are used to improve retention under constant aqueous flow. For example, magnetic targeting uses magnetic nanoparticles to load CRISPR components. Under an external magnetic field, these carriers can be guided toward the TM and accumulate circumferentially. This approach helps overcome the difficulty of achieving full TM coverage with conventional injection methods [125,126]. At the microscopic level, surface charge optimization enhances electrostatic interactions between carriers and the TM cell membrane, which improves local retention. Combined with PEGylation to improve stability, this strategy significantly increases the endocytic uptake of Cas9 mRNA in the aqueous humor environment [124]. Together, this combination of physical delivery routes and carrier engineering helps overcome delivery barriers at the trabecular meshwork. It provides a feasible therapeutic approach for POAG and other treatment-resistant forms of glaucoma.

### 5.3. Retinal Cells

#### 5.3.1. Retinal Ganglion Cells (RGCs)

Retinal ganglion cells (RGCs), located just beneath the inner limiting membrane (ILM), are the only output neurons in the retina that transmit visual signals to the central nervous system [127]. Their progressive degeneration is the pathological basis of blinding diseases such as glaucoma and Leber hereditary optic neuropathy (LHON) [128,129]. Currently, intravitreal injection (IVT) is the preferred administration route for gene therapy targeting RGCs. However, IVT delivery must overcome both the viscous barrier of the vitreous and the size-exclusion properties of the ILM. Therefore, successful gene editing for RGCs depends on the construction of vectors with strong diffusivity, penetrability, and cell-targeting capabilities. Although gene therapies targeting RGCs, especially CRISPR-based strategies using non-viral vectors, are still in early stages [130,131,132], exosomes have emerged as a promising delivery platform for overcoming these anatomical barriers. As membrane-derived natural vesicles, exosomes possess membrane fusion capabilities that enhance ILM penetration and retinal accumulation [133]. Further cationic surface modifications have been shown to improve their diffusibility in the vitreous, increasing the uptake efficiency by RGCs [134]. These findings suggest that engineered exosome-based platforms may provide a new non-viral strategy for CRISPR delivery to RGCs.

#### 5.3.2. Retinal Pigment Epithelium (RPE) and Photoreceptor Complex

For non-viral vectors administered via IVT to reach the outer retina, they must traverse the RGC layer and multiple intermediate neuronal layers, posing a significant delivery challenge. Anatomically, although the retinal pigment epithelium (RPE) and photoreceptors are separate layers, they form a closely integrated visual functional unit through the interdigitation of RPE microvilli and photoreceptor outer segments [135,136,137]. As they are typically co-targeted during subretinal injection (SRI), this section discusses them as a functional complex [138]. Photoreceptors include rods, responsible for scotopic vision, and cones, responsible for high-acuity and color vision. Rods are widely distributed and require high-coverage delivery systems, whereas cones are concentrated in the macula and demand highly specific targeting to avoid off-target effects in peripheral retina [139]. The RPE, located between the neurosensory retina and choroid, plays critical roles in photoreceptor support, phagocytosis, and the visual cycle. Its strong phagocytic activity may aid in the uptake of certain nanocarriers [136,140]. Dysfunction in any part of this RPE-photoreceptor unit can lead to hereditary blinding diseases such as retinitis pigmentosa (RP) and Leber congenital amaurosis (LCA) [141,142], often caused by loss-of-function mutations in genes such as RHO or CEP290. The therapeutic goal is to precisely correct mutations or restore gene function to rescue degenerative changes [143,144,145,146].

For non-viral CRISPR gene therapy targeting this region, SRI remains the most direct and effective approach for delivering gene-editing tools to the deep retina. By injecting carriers into the potential space between the RPE and photoreceptors, a localized drug reservoir with high concentration is created, although this procedure induces a controlled, iatrogenic retinal detachment [147,148]. Studies have shown that compacted DNA nanoparticles carrying therapeutic genes can transfect nearly all photoreceptors when delivered through this route [149]. In addition, VLP systems with surface modifications have been developed to achieve selective tropism toward RPE cells. After subretinal injection, these systems can deliver CRISPR/Cas9 ribonucleoprotein (RNP) complexes with efficiency comparable to that of AAV vectors [78]. However, despite its high transfection efficiency, SRI requires complex surgical procedures. The risks associated with its invasive nature, including retinal detachment and infection, cannot be ignored.

Because of these limitations, the development of non-invasive carriers that can be administered by IVT and penetrate the vitreous, inner limiting membrane, and deeper retinal layers remains a promising direction. To overcome this depth-related barrier, several engineered strategies have shown potential. For example, optimized LNP formulations delivered by IVT have been reported to cross retinal layers and selectively transfect RPE cells [150]. In another approach, a novel biopolymer–penetrating peptide system (Nuc1) delivered by IVT was able to transport recombinant proteins across the full thickness of the retina [151].

#### 5.3.3. Choroid and Suprachoroidal Space

The choroid is located between the RPE and the sclera. It is a highly vascularized tissue and supplies oxygen and nutrients to photoreceptors and the RPE [152,153]. CNV, caused by VEGF overexpression, is a key pathological basis of wet age-related macular degeneration (wAMD) [154]. Gene-editing approaches targeting VEGF therefore represent an important strategy for CNV treatment [155,156,157]. By using CRISPR/Cas systems to suppress VEGF expression or regulate other genes involved in angiogenesis, long-lasting therapeutic effects may be achieved [158,159,160,161]. At present, delivery of CRISPR components mainly relies on IVT and SRI. However, both routes are limited by intraocular barriers and by the need for high surgical precision [160,161].

In comparison, suprachoroidal space (SCS) injection has emerged as a potential alternative for choroidal therapy. This approach uses microneedle-based techniques to deliver carriers directly into the potential space between the sclera and the choroid. It allows precise delivery to choroidal tissue while maintaining a minimally invasive profile [162,163,164]. Currently, CRISPR gene-editing applications based on SCS delivery are still at an early stage, and their long-term in vivo safety and editing efficiency require further investigation.

Regardless of the delivery route, the development of engineered non-viral carriers with high penetration capacity and adaptability to the complex choroidal microenvironment remains critical for improving therapeutic outcomes [74,76]. In terms of microenvironment responsiveness, dynamic covalent lipid nanoparticles have been designed to respond to oxidative stress in CNV lesions. These systems undergo structural disassembly and release Cas9 mRNA only under high levels of reactive oxygen species (ROS), which reduces non-target toxicity [46]. To address limitations in cellular uptake, new biomimetic carriers such as engineered virus-like particles (Cas9-eVLPs) have been developed for efficient loading of RNP complexes. These systems have shown higher transfection potential than conventional liposomes [165]. In addition, active targeting strategies have been widely applied to improve recognition of vascular endothelial cells. For example, modification of LNP surfaces with RGD peptides enables specific binding to integrin receptors expressed on ocular neovascular tissue [166]. Alternatively, exosome–liposome hybrid nanoparticles combined with the nucleic acid aptamer AS1411 have also been used to target endothelial cells [95]. These strategies have been shown to enhance gene-editing efficiency and suppress pathological angiogenesis in CNV models.

## 6. Knowledge Gaps and Future Perspectives

### 6.1. Limitations of Current CRISPR/Cas9 Editing Mechanisms

The primary challenge bridging Cas9-mediated editing mechanisms and ocular gene therapy remains the risk of off-target effects, which manifests on both genomic and spatial levels [167]. Genomically, it is established that the prolonged expression of Cas9 increases the incidence of off-target editing [168]. Unfortunately, key therapeutic targets in the eye, such as the corneal endothelium, trabecular meshwork, photoreceptors, and RPE, are predominantly post-mitotic cells. Such intracellular off-targeting leads to unintended cleavage at non-target DNA loci, potentially inducing tumorigenesis [7]. To mitigate these risks, high-fidelity SpCas9 variants, including eSpCas9, Cas9-HF1, HypaCas9, and SniperCas9, are being rigorously investigated to minimize non-specific genome interactions and limit off-target mutations [169,170]. Spatially, the complex anatomy of the eye necessitates precise delivery control. While molecular cargo delivery can be tailored via specific injection routes to target specific tissues, the risk of vector diffusion to unintended intraocular structures persists. Consequently, beyond optimizing the editor itself, engineering non-viral carriers represents a critical strategy to enhance tissue-specific targeting and overcome these spatial limitations.

### 6.2. Bottlenecks in Non-Viral Delivery

Although non-viral vectors demonstrate promising potential for CRISPR delivery, as discussed previously, they currently remain predominantly in laboratory or preclinical stages due to the inherent limitations of various carrier strategies. To overcome these limitations, the convergence of Machine Learning (ML) and generative AI is revolutionizing nanocarrier optimization. By integrating molecular docking and multi-omics analysis, researchers may be able to employ AI-assisted design to engineer nanoparticles that selectively target specific ocular tissues [171,172]. Another approach is the development of stimuli-responsive carriers. These responsive systems enable spatiotemporal control by reacting to internal (e.g., pH, ATP and ROS) or external (e.g., light, ultrasound) triggers, thereby confining therapeutic activation to the target site and minimizing off-target effects [173].

### 6.3. Safety Concerns and Clinical Translation

As non-viral CRISPR therapeutics advance toward clinical trials, the primary focus of preclinical evaluation is biosafety and translational feasibility. Current safety assessments face challenges regarding standardization and rely predominantly on rodent models that, due to metabolic differences, may not fully predict human physiological outcomes [173]. Consequently, future research underscores the necessity of comprehensive and long-term preclinical studies in non-human primate models for rigorous risk evaluation. Key safety considerations include the potential adaptive immune response to Cas9 proteins [167,174], potential neurotoxicity or functional impairment associated with the accumulation of inorganic delivery systems in the retinal pigment epithelium (RPE) and trabecular meshwork [175], and the need to rigorously evaluate the safety profile of emerging bio-inspired delivery systems, such as exosomes, regarding specific risks like potential carcinogenicity [96].

Parallel to these biological hurdles is the challenge of achieving manufacturing scalability. Adapting laboratory-scale protocols to Good Manufacturing Practice (GMP) standards remains a critical bottleneck, as traditional methods often result in unacceptable batch-to-batch heterogeneity [176,177], nanoparticle aggregation during sterile filtration, and mRNA degradation under cryopreservation conditions [178]. While current purification techniques can compromise the structural integrity of bio-nanoparticles [179]. Finally, the economic implication is significant; the complexity and limited yield of current fabrication methods contribute to elevated production costs. Consequently, there is a pressing need to innovate fabrication methodologies to ensure the high purity, consistency, and stability required for clinical use, alongside the cost-effectiveness necessary for widespread patient access.

## 7. Conclusions

The advent of CRISPR/Cas9 technology has revolutionized the landscape of ocular gene therapy, offering a transformative approach to treating blindness at its genetic root. As highlighted in this review, non-viral delivery systems, which range from versatile lipids and polymers to robust inorganic carriers, have emerged as safer alternatives to viral vectors, effectively overcoming limitations such as restricted cargo capacity and insertional mutagenesis. Although challenges regarding in vivo delivery efficiency and long-term biocompatibility persist, the rapid convergence of rational carrier design, microenvironment-responsive strategies, and advanced gene editing tools is paving the way for precise ocular interventions. As these technologies mature and bridge the translational gap, non-viral CRISPR therapeutics are poised to evolve from experimental concepts into standard-of-care treatments for patients with hereditary and refractory ocular diseases.

## Figures and Tables

**Table 1:** A comprehensive overview of current and past clinical trials involving CRISPR system in ocular gene therapy.

ClinicalTrials.gov ID	Intervention Name	Vector/Delivery Platform	Target Gene	Editing System	Condition	Administration Route
NCT05805007	ZVS203e	Recombinant AAV (rAAV)	RHO	CRISPR/Cas9	Retinitis Pigmentosa	Subretinal injection
NCT06465537	BD113	Virus-Like Particle (VLP) (Lentivirus-derived, Integration-defective)	MYOC	CRISPR/Cas9	POAG	Intracameral injection
NCT04560790	HELP (BD111)	Lentiviral Particle (mLP) (mRNA-carrying)	UL8 & UL29 (HSV-1)	CRISPR/Cas9	Herpes Simplex Keratitis	Intrastromal injection
NCT03872479	EDIT-101	Recombinant AAV5 (rAAV5)	CEP290	CRISPR/Cas9	LCA10	Subretinal injection
NCT06623279	HG202	Recombinant AAV (rAAV)	VEGFA	CRISPR/Cas13 *	nAMD	Subretinal injection
NA	GEB-101	engineered protein delivery vehicle (PDV)	TGFBI	CRISPR/Cas9	TGFBI corneal dystrophy	Intrastromal injection

Source: Data were primarily retrieved from ClinicalTrials.gov. Information for the GEB-101 trial was retrieved from the GenEdit Bio official announcement (https://www.geneditbio.com/newsinfo/8412946.html (accessed on 16 February 2026)). * Note: HG202 utilizes the Cas13 system to target RNA, representing a new direction in ocular gene editing applications—specifically, post-transcriptional regulation without altering genomic DNA. Therefore, it is retained in this overview.

**Table 2:** Comparison of non-viral CRISPR/Cas9 delivery systems discussed in this review.

Delivery System	Advantages	Limitations
Lipid Nanoparticles (LNPs)	High payload capacity. Good biocompatibility. pH-responsiveness.	Potential toxicity. Poor storage stability. Limited targeting specificity.
Polymer Nanoparticles (PNPs)	Good biocompatibility. Good biodegradability. Easy to chemically modify. Specificity of biopolymers.	Potential cytotoxicity. Variable transfection efficiency among types. Complex in production.
Inorganic Nanoparticles (INPs)	Wide variety of materials. Responsive to physical stimuli like light and magnetic fields. High stability in physiological environments.	Poor biodegradability: Risk of long-term accumulation. Unclear long-term toxicity.
Virus-Like Particles (VLPs)	High transfection efficiency. Fast expression kinetics. Low frequency of off-target mutations.	May still trigger immune response. Limited cargo space. Complex production and purification processes.
Exosome-Based Nanoparticles (Exo-NPs)	Good biocompatibility. Low immunogenicity. Ability to cross biological barriers.	High batch heterogeneity. Difficult purification and hard to scale up. Generally low loading efficiency for cargo.

## Data Availability

The original contributions presented in this study are included in the article.

## Abbreviations

AAV	Adeno-associated virus
BRB	Blood–retinal barrier
Cas9	CRISPR-associated protein 9
CEC	Corneal endothelial cell
CNV	Choroidal neovascularization
CPP	Cell-penetrating peptide
CRISPR	Clustered regularly interspaced short palindromic repeats
DSB	Double-strand break
ECM	Extracellular matrix
EDCas9	Enhanced-deletion Cas9
FECD	Fuchs endothelial corneal dystrophy
GNP	Gold nanoparticle
HDR	Homology-directed repair
HNP	Hybrid nanoparticle
HSK	Herpes simplex keratitis
IC	Intracameral injection
ICAM-1	Intercellular adhesion molecule-1
ILM	Inner limiting membrane
IOP	Intraocular pressure
IVT	Intravitreal injection
LCA	Leber congenital amaurosis
LHON	Leber hereditary optic neuropathy
LNP	Lipid nanoparticle
MECD	Meesmann epithelial corneal dystrophy
MRI	Magnetic resonance imaging
MSN	Mesoporous silica nanoparticle
NHEJ	Non-homologous end joining
PCL	Polycaprolactone
PEI	Polyethyleneimine
PLGA	Poly(lactic-co-glycolic acid)
PNP	Polymer nanoparticle
POAG	Primary open-angle glaucoma
RGC	Retinal ganglion cell
RNP	Ribonucleoprotein
ROS	Reactive oxygen species
RP	Retinitis pigmentosa
RPE	Retinal pigment epithelium
SCS	Suprachoroidal space
sgRNA	Single-guide RNA
SRI	Subretinal injection
TM	Trabecular meshwork
VEGF	Vascular endothelial growth factor
VLP	Virus-like particle
wAMD	Wet age-related macular degeneration
